# Magnetic Nanoparticles Coated with a Thermosensitive Polymer with Hyperthermia Properties

**DOI:** 10.3390/polym10010010

**Published:** 2017-12-22

**Authors:** Felisa Reyes-Ortega, Ángel V. Delgado, Elena K. Schneider, B. L. Checa Fernández, G. R. Iglesias

**Affiliations:** 1 Department of Applied Physics, University of Granada, Av. Fuentenueva s/n, 18071 Granada, Spain; adelgado@ugr.es (Á.V.D.); lunachecaf@gmail.com (B.L.C.F.); iglesias@ugr.es (G.R.I.); 2 Drug Delivery, Disposition and Dynamics, Monash Institute of Pharmaceutical Sciences; Monash University, Parkville, Victoria 3052, Australia; elena.schneider@unimelb.edu.au

**Keywords:** hyperthermia, magnetite nanostructures, polymer coating, temperature-responsive polymer

## Abstract

Magnetic nanoparticles (MNPs) have been widely used to increase the efficacy of chemotherapeutics, largely through passive accumulation provided by the enhanced permeability and retention effect. Their incorporation into biopolymer coatings enables the preparation of magnetic field-responsive, biocompatible nanoparticles that are well dispersed in aqueous media. Here we describe a synthetic route to prepare functionalized, stable magnetite nanoparticles (MNPs) coated with a temperature-responsive polymer, by means of the hydrothermal method combined with an oil/water (o/w) emulsion process. The effects of both pH and temperature on the electrophoretic mobility and surface charge of these MNPs are investigated. The magnetite/polymer composition of these systems is detected by Fourier Transform Infrared Spectroscopy (FTIR) and quantified by thermogravimetric analysis. The therapeutic possibilities of the designed nanostructures as effective heating agents for magnetic hyperthermia are demonstrated, and specific absorption rates as high as 150 W/g, with 20 mT magnetic field and 205 kHz frequency, are obtained. This magnetic heating response could provide a promising nanoparticle system for combined diagnostics and cancer therapy.

## 1. Introduction

The joint efforts of nano-pharmacy and targeted drug delivery have resulted in a number of designs of nanoscale (notably, magnetic) materials, susceptible to be used as drug delivery carriers. These magnetic nanoparticles (MNPs), based on ferromagnetic elements, such as iron, nickel, cobalt, and their compounds (ferrites, and specially magnetite and maghemite are perhaps the best representatives of the group, in terms of the frequency of their use and their rather low toxicity). Their applications in the diagnosis and treatment of diseases, and very specifically of cancer, have grown exponentially during the last decade [1,2,3,4,5,6,7,8,9,10,11,12,13,14,15]. MNPs represent perhaps one of the most versatile groups of materials and, as such, intensive research efforts have focused on the development of appropriate protocols for their synthesis with well-defined, uniform size and shape in a reproducible manner. In order to be efficient in biological applications, colloidal suspensions of magnetic particles must have long-time stability in aqueous media, and their magnetic core must respond to an external magnetic field so as to eventually drive the particles to a desired location and maintain them there.

The synthesis of these and related smart nanomaterials, which can respond to the surrounding stimuli, is considered a potent tool in biomedicine. For instance, temperature-responsive MNPs have been previously designed by incorporating magnetic cores into a thermo-sensitive polymer, such as poly(*N*-isopropylacrylamide) (PNIPAAm) [16,17]. Another thermo-sensitive polymer less widely used in the literature, but with very interesting properties due to its dual sensitive response (temperature and pH), is poly(2-(dimethylamino)ethyl methacrylate) (PDMAEMA) [18]. They both show water-soluble and hydrophilic properties below the low critical solution temperature (LCST), while above this temperature they undergo a sharp coil-to-globule transition that results in hydrophobic and insoluble aggregates [19,20]. Furthermore, the PDMAEMA quaternary ammonium salt, used in this work, shows extra advantages in biomedicine due to its antimicrobial activity [21], aqueous media compatibility [22], and DNA conjugates applications [23]. The combination of a stimuli-responsive polymer with magnetic nanoparticles in a single system may result in a compelling drug delivery system with controlled release and better accumulation within solid tumors, providing a homogeneous spatial distribution in tumor tissues, and increasing the intracellular localization of anticancer therapeutics.

As an additional feature of these systems, they can be used in magnetic hyperthermia, a suitable technology aimed at terminating malignant cancers by using MNPs that generate heat in response to the application of an external alternating magnetic field (AMF). It is needed to obtain MNPs with optimal heating characteristics that allow the clinical application of hyperthermia technology in a safe manner, while being effective in cancer treatment. In particular, the AMF itself must be safe and well tolerated. In clinical terms, this means that the magnetic field strength and frequency must be sufficiently low as to elude the induction of eddy currents strong enough to generate harmful levels of non-specific heating in tissues, or of peripheral nerve stimulation [24]. Thus, it is clinically preferred a particle with lower requirements regarding field strength or frequency, but showing the best possible heating properties for a given AMF strength and frequency.

The fundamental explanation for the hyperthermia response of MNPs is the lag between the magnetization and the field when the particles are exposed to alternating fields of suitable frequency. Only hysteresis loss and Brownian and Néel relaxations can be the possible mechanisms to explain this behavior, since eddy current losses are negligible below the GHz frequency range if the particle size is at or below 100 nm [1,2]. If the particles are multidomains, the heat evolved comes from the hysteresis of their magnetization. When particles are monodomains, the power is not related to hysteresis loops, but rather, it is associated to Brownian and Néel relaxations [25]. For the sought application of magnetic heating of tissues, the quantity of interest is the specific absorption rate (SAR) that is defined as the thermal power dissipated per unit mass of material in the sample subjected to the AMF [25].

In this paper, we analyzed the use of hydrothermal synthesis for obtaining initially hydrophobic magnetite particles. The essence of the hydrothermal method lies in the heating of the metal oxide precursor in solution or suspension at an elevated temperature and pressure (300 °C, 10 MPa). The solubility of substances increased with increasing temperature; the precipitation of the reaction product proceeded more slowly, and the produced crystals were smaller than after precipitation under conventional conditions. The particles were subsequently transferred to aqueous media and coated with PDMAEMA in order to make them biocompatible and stable in aqueous media at physiological pH. To our knowledge, there is no previous work describing procedures for directly coating the magnetic nanoparticles using a temperature- and pH-sensitive polymethacrylate (PDMAEMA) with a simple emulsion step. Published works [26,27,28,29] describing a polymer-coated MNP system involve a polymerization step or covalent grafting reaction, which includes the use of initial reactants (monomers, initiators, catalysts, etc.), the need of an additional purification step to remove the non-reacted reagents, longer preparation time, involved synthesis conditions (vacuum, inert atmosphere, high temperatures, etc.). In this work, in addition to characterizing the particles in terms of size and surface properties (electrokinetic potential), before and after the coating process, their heating capacity was studied and checked as potentially therapeutic hyperthermia systems. Specific absorption rate (SAR) and intrinsic loss power (ILP) were determined with this purpose.

## 2. Materials and Methods

### 2.1. Materials

All reactants used were commercially available: Hexadecylamine (HDA) 98% purity, oleic acid (OA) 99% purity, iron pentacarbonyl, dibenzyl ether 98% purity, poly(2-dimethylamino)ethyl methacrylate) methyl chloride quaternary salt (PDMAEMA), tetramethylammonium hydroxide (TMAOH) solution 25 wt % in water, and polyvinyl alcohol (PVA) 99% hydrolyzed and average M_w_ (89,000–98,000) g/mol, were purchased from Sigma-Aldrich (St. Louis, MO, USA) and were used as received. Absolute ethanol and n-hexane 99% reagent grade were supplied by Scharlau (Munich, Germany).

### 2.2. Methods

#### 2.2.1. Synthesis of the Fe_3_O_4_ Nanoparticles

Iron oxide nanoparticles were synthesized using a modification of a previously described hydrothermal method [4,30], performed as follows: 2.54 mmol (0.6 g) HDA and 25.21 mmol (8 mL) OA were mixed in 16 mL benzyl ether. This mixture was heated up to 55 °C and stirred for 30 min. After that, the solution was cooled to room temperature and 30.42 mmol (4 mL) of iron pentacarbonyl was added and magnetically stirred for 60 min. Then, the solution was transferred to a 160 mL autoclave with a Teflon lining and heated to 300 °C for 6 h. After reaction in the autoclave, the mixture is cooled to room temperature, and a black, magnetic precipitate was obtained. The product of the hydrothermal synthesis (Fe_3_O_4_ MNPs) is separated from the mother liquor, followed by repeated washing with ethanol, and finally dried or re-suspended in hexane.

#### 2.2.2. Phase Transfer of Fe_3_O_4_ Nanoparticles to Aqueous Media

5 mL of the obtained MNPs suspension in hexane (20 mg/mL) were dispersed in 5 mL of distilled water containing 500 µL of TMAOH and 500 µL ethanol. This mixture was sonicated with an ultrasound probe (Branson Sonifier 450, Fisher Scientific, Hampton, NH, USA) during 10 min. The organic phase was separated from the aqueous medium using a 100 mL separatory funnel. This transfer procedure was repeated three times to make sure that the hexane solvent was completely removed and particles could then be dispersed in a pure aqueous medium. The TMAOH aqueous dispersion (pH = 12) was adjusted to a pH value of 7.4 by adding a suitable amount of HNO_3_ (0.1 M).

#### 2.2.3. Preparation of PDMAEMA-Coated Fe_3_O_4_ Magnetic Nanoparticles

The preparation of coated nanoparticles was carried out by a simple emulsion step: 3 mL of ethyl acetate Fe_3_O_4_ suspension (10 mg/mL) was first emulsified by the addition of 9 mL of the aqueous PDMAEMA solution (1% *w*/*v*) containing PVA (2% *w*/*v*). Then ethyl acetate was removed using a rotary evaporator at 60 °C and 7 mbar. Coated MNPs were then magnetically decanted, washed, and re-suspended in water three times.

#### 2.2.4. Characterization of the Samples

Electrophoretic mobility measurements were carried out in a Zetasizer Nano-ZS (Malvern Instruments, Worcestershire, UK). In the case of suspensions with various pH values, they were prepared by simply adding 0.5 mL of magnetite suspensions (10 mg/mL) to 50 mL 5 mM KNO_3_ (fixed ionic strength) until finally obtaining a slightly turbid solution adequate for this type of determination. The pH value of the suspensions was then adjusted by adding a suitable amount of KOH (0.01 or 0.1 M) or HNO_3_ (0.01 or 0.1 M). For each suspension, five measurement runs were taken, with 11 iterations in each run. The zeta potential, *ζ* was obtained from the O’Brien and White electrophoresis theory [31]. Particle size measurements were carried out using the same apparatus.

The morphology of the nanoparticles was analyzed by transmission and scanning electron microscopy (TEM and SEM) using a LIBRA 120 Plus Carl Zeiss microscope (Oberkochen, Germany) and a Hitachi S-510 (Tokyo, Japan), respectively. FTIR spectra were obtained on a JASCO 6200 FT-IR (Tokyo, Japan) spectrometer with SPECTRA MANAGER V2 software (JASCO Analitica Spain S.L., Madrid Spain). Samples were prepared by grinding 1–1.5 mg of dry particles with 150 mg of potassium bromide powder and pressing the mixture with a pellet-forming die. A force of approximately 10^5^ N was applied under a vacuum of several mm Hg for several minutes to form transparent disks. These were then analyzed without further treatment at room temperature with 50 scans, and at a resolution of 4 cm^−1^.

The crystal phase was identified by recording X-ray powder diffraction patterns (XRD) of the dry, washed samples using a Bruker D8 Advance diffractometer (Berlin, Germany) equipped with a Cu Kα radiation source (λ = 1.5406 Å) and a Bruker LINXEYE detector. Analysis was carried out at 25 °C, 40 kV and 40 mA. The 2θ measured range was 17°–70°, at 0.02° step, with a measurement time of 575.7 s/step.

Thermogravimetric analysis (TGA) was performed on a TGA-50H SHIMADZU (Kyoto, Japan) device with vertical oven and a maximum precision of 0.001 mg. Samples were analyzed under 50 mL/min nitrogen flow, and at a heating rate of 10 °C/min, from 30 to 950 °C.

Magnetization cycles were obtained at room temperature in an MPMS-XL SQUID magnetometer (Quantum Design, San Diego, CA, USA). Between 1 and 3 mg dried samples were used for these measurements.

Hyperthermia measurements were performed by placing 0.5 mL of each sample in a cylindrical glass tube (0.9 cm internal diameter) and recording the temperature as a function of time with the magnetic field on. The hyperthermia assays were carried out using a homemade ac current generator based on a Royer-type oscillator set at different frequencies in the range 100–200 kHz, with maximum current of 8 A. The current is passed through an eight turn coil, 20 mm in diameter and 45 mm in length, made of copper tube. The amplitude of the magnetic field induction was 20.25 mT in the center of the coil, measured with a NanoScience Laboratories Ltd., Probe (Newcastle, UK), with a 10 μT resolution. The temperature of the samples was registered at 3 s intervals with a fiber optic thermometer (Optocon AG, Dresden, Germany). The experiments were always performed in samples pre-heated at 25 °C.

From the initial slope dT/dt (first 30 s of the experiment) of the temperature vs. time data, the specific absorption rate (SAR; also known as specific loss power SLP) of the suspensions of MNPs was calculated as follows (1):

(1) $$\mathrm{SAR}=\frac{CV_{s}}{m}\frac{dT}{dt}$$

where *C* is the volume specific heat capacity of the sample ($C_{H_{2}O}=$ 4185 J/LK, $C_{\mathrm{hexane}}=$ 1502 J/LK), *V_s_* = 0.5 mL is the sample volume, and *m* is the mass of dispersed particles. The concentration of solids in the tested suspensions was 10 mg/mL. The heating power as evaluated by the SAR is determined not only by the particle properties, but also by the field strength *H*_0_ and its frequency *f*, with quadratic and linear dependences, respectively. These extrinsic components of the absorption rate can be eliminated to obtain the so-called intrinsic loss power (ILP), given by (2):

(2) $$\mathrm{ILP}=\frac{\mathrm{SAR}}{fH_{0}^{2}}$$

It should be remarked that, due to the field and frequency dependence of the complex susceptibility of the MNPs, the ILP parameter can only be considered constant in relatively low field strength and low frequency regimes. The practical units used for SAR are W/g, and for ILP the unit is nH·m^2^/kg (SAR in W/kg, *f* in kHz and *H*_0_ in kA/m).

## 3. Results and Discussion

### 3.1. Morphology and Particle Size Distribution of the Coated Nanoparticles by TEM and DLS

**Figure 1:**
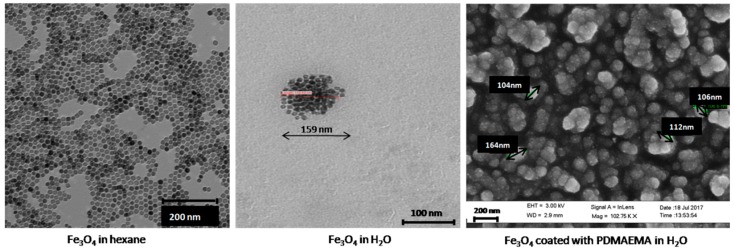
Transmission electron microscopy (TEM) images of Fe_3_O_4_ nanoparticles in hexane (**left**), and water (**center**) before coating. Scanning electron microscopy (SEM) picture after coating with poly(2-(dimethylamino)ethyl methacrylate) (PDMAEMA) (**right**).

**Figure 2:**
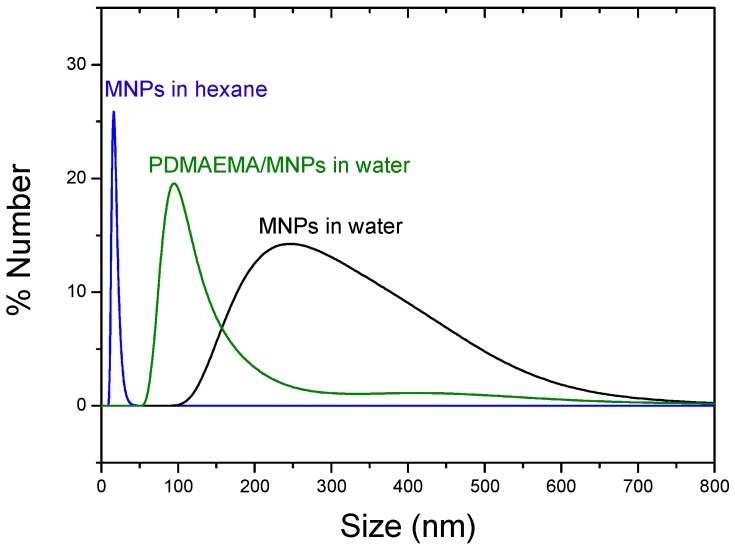
Particle size distributions by number of Fe_3_O_4_ nanoparticles in the indicated conditions.

TEM images in Figure 1 show the high sphericity and homogeneity of the Fe_3_O_4_ nanoparticles. Their mean diameter was measured using DLS, obtaining a mean diameter (±S.D.) of 17.2 ± 0.1 nm when dispersed in hexane (PDI = 0.05). The resulting nanoparticles were very stable against agglomeration in organic medium because of the surfactant molecules (oleic acid) attached to the surface of the magnetic cores. After transferring them to the aqueous medium, spherical aggregates were formed, about 250 ± 25 nm average diameter (PDI = 0.32). The PDMAEMA-coated particles formed irregular quasi-spherical aggregates with internal structure consisting of a polymer envelope containing the MNPs, with a mean diameter around 98 ± 20 nm (PDI = 0.39). Particle size distributions were analyzed by DLS and compared with the electron microscope pictures, observing a good agreement between the average sizes shown by both methods (Figure 2), although sizes observed by DLS were slightly larger, as some aggregation in the aqueous medium could not be discarded. As observed, single nanoparticles were well separated in hexane due to their hydrophobic nature.

### 3.2. Physicochemical Characterization

**Figure 3:**
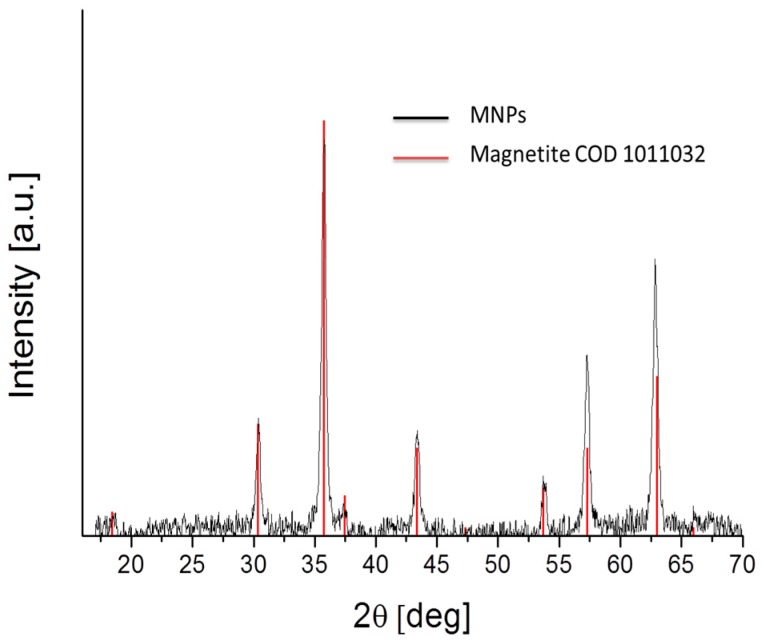
X-ray powder diffraction (XRD) diffraction pattern of Fe_3_O_4_ synthesized nanoparticles. The vertical lines are the magnetite pattern (COD 1011032).

The iron oxide phase of the synthesized nanoparticles was identified from the XRD pattern, as shown in Figure 3, with peak positions at 30.4 (220), 35.8 (311), 37.2 (222), 43.5 (400), 53.9 (422), 57.5 (511) and 63.1 (440) degrees, which are consistent with the standard data for magnetite (COD1011032).

**Figure 4:**
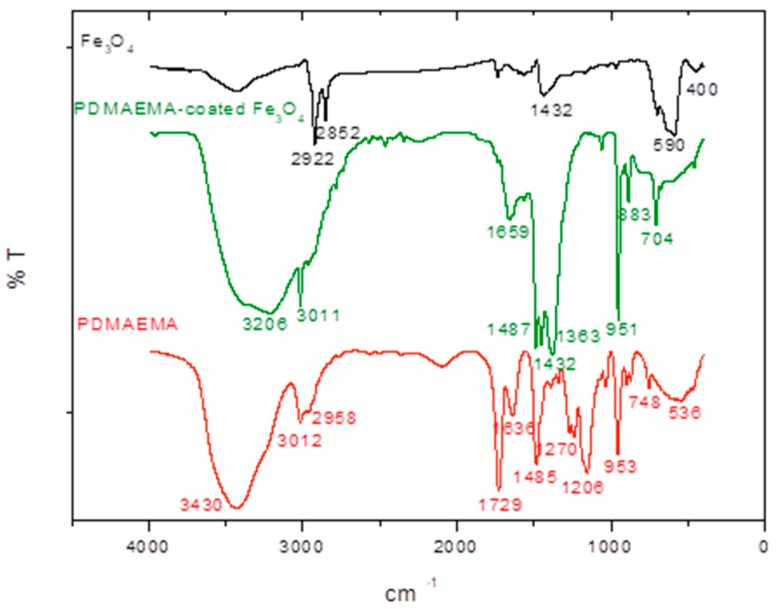
FTIR spectra of Fe_3_O_4_ particles, PDMAEMA and PDMAEMA-coated Fe_3_O_4_ particles.

According to the FTIR spectra (Figure 4), the magnetic coated nanoparticles (Fe_3_O_4_-PDMAEMA) showed the characteristic bands of pure magnetite at around 590 and 400 cm^−1^ corresponding to the Fe–O/Fe–O–Fe bonds [32], and the C=O stretching band at 1729 cm^−1^, the C–N stretching band at 1636 cm^−1^ and N-H stretching bands at 3430 cm^−1^ of the amino- bonds belonging to the quaternary PDMAEMA salt, and the C–O stretching bands of this polymer at 1485 cm^−1^, slightly shifted to a lower wavelength (1659, 1561, 3206, 1448 cm^−1^ respectively) due to the hydrogen bonds and electrostatic forces created between the magnetic core and the polymer shell.

**Figure 5:**
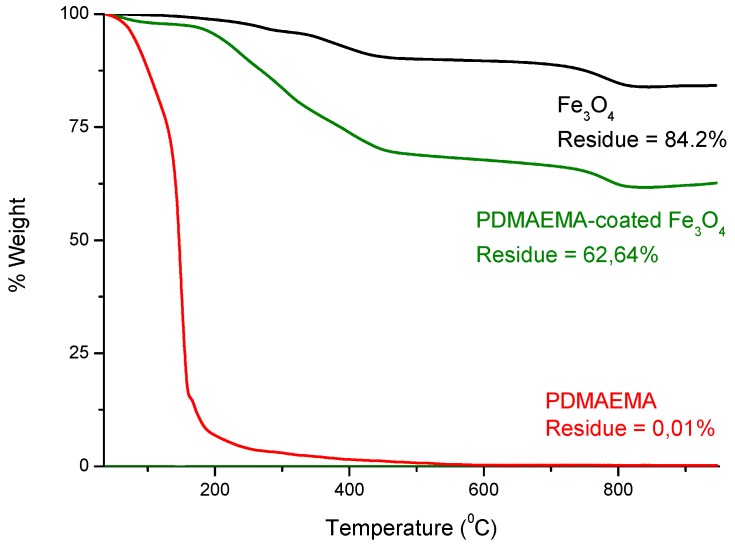
Thermograms of the magnetic nanoparticles.

In addition, thermogravimetric analyses were used to evaluate the thermal stability of the magnetic particles, as well as to check the amount of organic/magnetic composition on the functionalized nanoparticles. The thermograms of the magnetic nanoparticles before and after PDMAEMA coating are shown in Figure 5. The weight loss curve recorded from the TGA analysis of the Fe_3_O_4_-PDMAEMA system showed two well-resolved degradation steps, which allowed a quantitative evaluation of the composition, taking into account the total weight loss percentage and the residue left after thermal degradation. The Fe_3_O_4_-PDMAEMA weight percentages were determined from the TGA curves by considering those corresponding to the original components (Fe_3_O_4_ and PDMAEMA). It was found that the amount of Fe_3_O_4_ in the nanoparticles is 74 wt %, with a 26 wt % of PDMAEMA shell coating.

### 3.3. Zeta Potential of the Magnetite Particles

**Figure 6:**
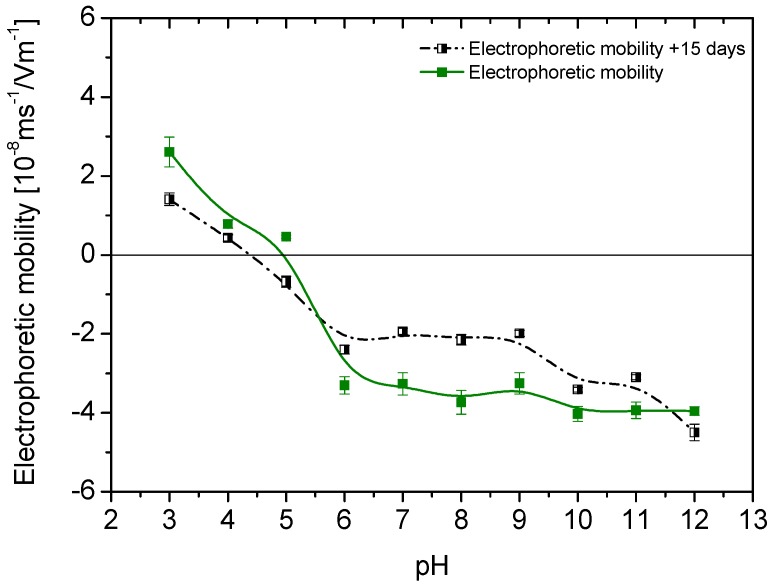
Electrophoretic mobility vs. pH for magnetite immediately after phase transfer to water, and after 15 days stored at room temperature in aqueous medium.

When nanoparticles are transferred to the aqueous medium, one can expect that they will acquire surface charge as a result of the ionization of the adsorbed TMAOH used for phase transfer. Since this is a strong base, OH– will be released in aqueous solution, leaving positive cations adsorbed. However, since it is well known that the presence of hydroxyls in solution produces an increase in the negative charge of magnetite particles (the isoelectric point [33] of this oxide is around pH_iep_ = 7), if these are not completely covered by N(CH_3_)_4_^+^, we can expect some balance of the positive effect of this cation and the negative contribution of pH variations associated to TMAOH. The results in Figure 6 (which in addition demonstrate the stability of the treatment, as the electrophoretic mobility remains practically unchanged after 15 days in water) show that this is indeed the case, and that magnetite shows an isoelectric point only slightly lower than the hydrophilic one.

**Figure 7:**
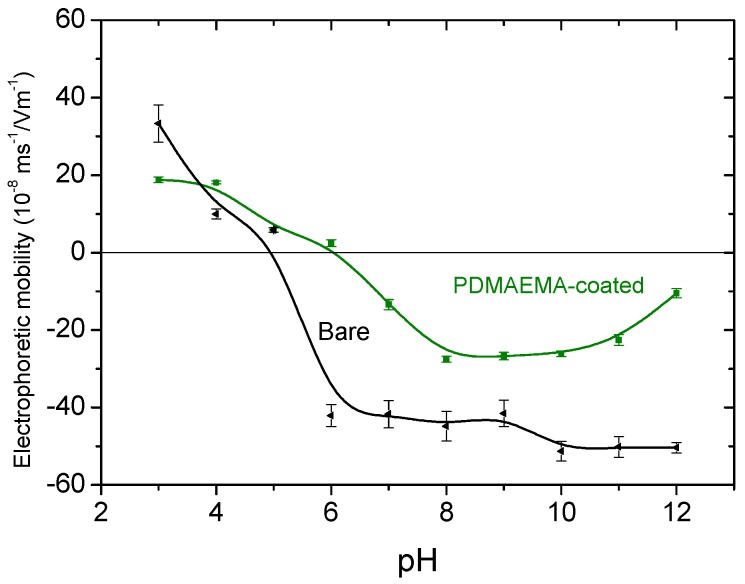
Effect of pH on the electrophoretic mobility of magnetite nanoparticles before and after PDMAEMA coating.

Similarly, the adsorbed PDMAEMA, also ionizable, can be expected to produce electrophoretic mobility changes, and it will determine the net charge of the adsorbed molecules, and hence of the coated particles, as long as the magnetite nanoparticles are completely hidden from the medium. From the observations in Figure 7, this might not be strictly the case, as the particles can be at least partially exposed to the aqueous solution. As before, electrophoretic mobility measurements can help us to identify the pH dependence of the surface charge of the coated particles, and eventually obtain some information regarding the predicted stability of the system in water. Figure 7 shows the results: at pH > 5.3 the synthesized nanoparticles have a negative charge, thus favoring the adsorption of PDMAEMA molecules. The polymer coating was carried out near pH 7, a value indicated as suitable to obtain the maximum chemisorption of the adsorbent. As observed in Figure 7, the isoelectric point of the Fe_3_O_4_-PDMAEMA system shifted to 6.2, higher than that of the hydrophilized magnetite nanoparticles, due to the neutralization of the negative surface charge by the positive charges (–N^+^(CH_3_)_3_) along the polymer chains.

**Figure 8:**
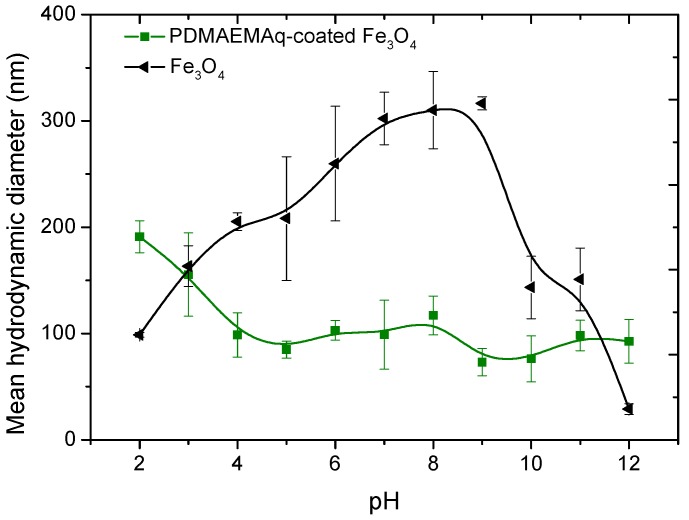
Effect of pH on the particle size of magnetite nanoparticles before and after PDMAEMA coating.

As mentioned, an essential aspect of the produced nanostructures is their stability. In situ particle size determinations by dynamic light scattering are shown in Figure 8. Rapid flocculation leading to the subsequent formation of large aggregates was observed for the bare Fe_3_O_4_ dispersion within the pH range 5–10. Apparently, the charge of the TMAOH molecules was too low at this interval for providing stability, and it was likely that the particles still had traces of oleate, and hence hydrophobic patches leading to aggregation. Interestingly, this aggregation did not take place when the particles were coated by PDMAEMA: this polymer could modify the size of the resulting nanostructures in function of the changes in the pH of the surrounding environment: as mentioned, the charged moieties of PDMAEMA consist of (–N^+^(CH_3_)_3_) groups, which would be more easily adsorbed onto magnetite around or above its pH_iep_ (Figure 7), this explained that the coated particles were largely stable above pH 5 (Figure 8), when the MNPs were expected to be efficiently coated by PDMAEMA.

### 3.4. Temperature Effects

**Figure 9:**
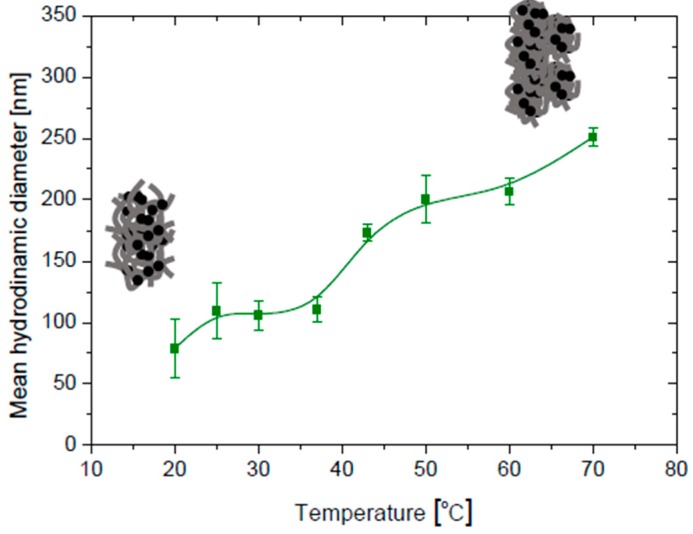
Effect of temperature on the hydrodynamic diameter of PDMAEMA-coated Fe_3_O_4_ aqueous suspension at pH 7.

Recall that at the LCST the temperature is such that the polymer solution undergoes phase separation from the isotropic state to an anisotropic one with two coexisting phases, one rich and one poor in polymer. Below the LCST, the enthalpy term, related to the hydrogen bonding between the polymer and the water molecules, is responsible for the polymer dissolution. When the temperature is raised above the LCST, the entropy term (hydrophobic interactions) dominates leading to polymer precipitation. It has been demonstrated [34] that the quaternization of this polymer increases its hydrophilic character, and therefore the LCST is shifted toward higher temperatures as more energy is required in these systems to break the more stable interactions between the new added cationic species in the polymer and water molecules [35]. Hence, as shown in Figure 9, when temperature is increased, the average size of the polymer envelope increases by aggregation of already existing magnetite/polymer composites, as such aggregation is favored by the increased hydrophobicity of the PDMAEMA envelope.

**Figure 10:**
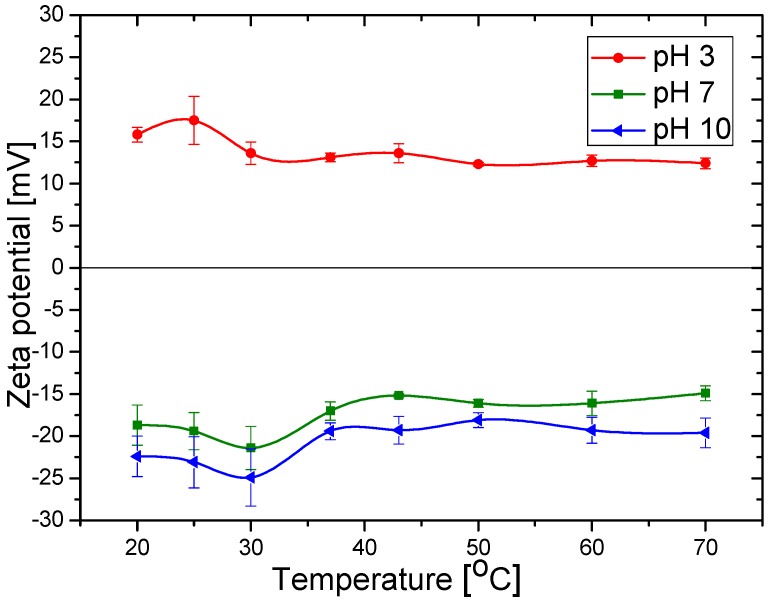
Effect of pH and temperature on the zeta potential of Fe_3_O_4_ particles coated with PDMAEMA.

The effect of temperature on the zeta potential of coated particles at selected pH values is shown in Figure 10. It can be observed that the potential decreases slightly in absolute value when temperature increases. It is likely that when the LCST temperature is approaching, hydrated ions are expelled from the polymer shell and the ionic strength of the surrounding medium increases to some extent, producing the decrease in the absolute zeta potential value with temperature, whatever the pH.

## 4. Magnetization and Hyperthermia Response

**Figure 11:**
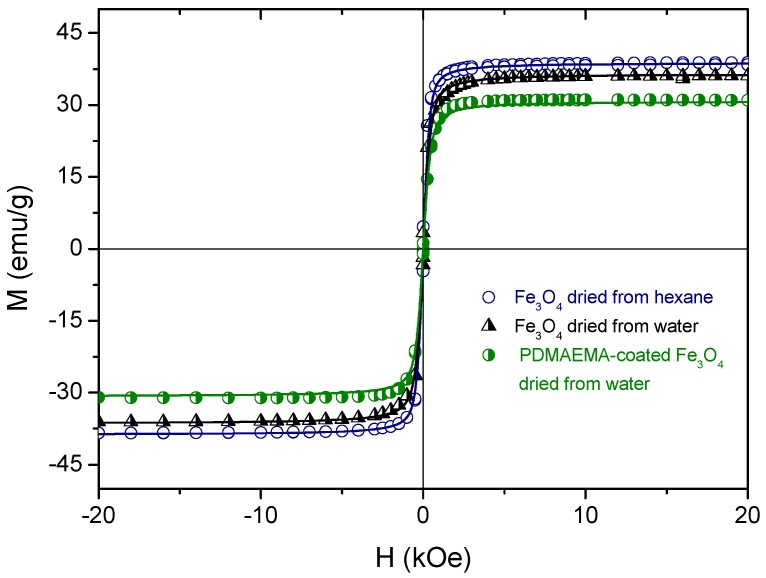
Magnetization curves of bare and coated magnetite nanoparticles. The lines are the best fits to the Langevin equation.

Both bare and coated Fe_3_O_4_ nanoparticles were first characterized in terms of their magnetic properties (Figure 11). The magnetization values have been normalized to the total mass of the sample. It can be observed that for all the samples the magnetization of Fe_3_O_4_ tends to saturate at approximately 1000 Oe, and no noticeable coercivity or remanence are observed. It is also observed an absence of hysteresis in all the magnetization curves, which indicates the superparamagnetic response of these systems. The curves were fitted to the Langevin equation [36,37] in order to confirm the superparamagnetic character of our samples (*m* is the magnetic moment per particle):

(3) $$\frac{M}{M_{s}}=\coth\xi -\frac{1}{\xi },\;\xi =\frac{\mu_{0}mH}{k_{B}T}$$

The results of the fittings are superimposed as solid lines on the experimental data points in Figure 11; note the agreement between them. From these fittings, we could estimate the values of *m* for each of the three kinds of particles: 4.64 ± 0.05, 3.4 ± 0.1, 3.3 ± 0.2 (10^−19^ Am^2^). Note the reduction in magnetic moment and in saturation magnetization upon either treatment to make the particles water dispersible or coating them with the polymer. Such reductions with respect to the bare MNPs are due to the non-magnetic contribution of the stabilization layers, although the differences are not very significant.

It must be noted that the saturation magnetization of these MNPs is lower than the bulk magnetite value (90 emu/g, see, e.g., [38]). The hydrothermal method used for their preparation and the resulting crystallinity play an essential role in this value, and in fact lower saturation (around 50 emu/g) has been reported for spherical magnetite nanoparticles [30], a fact attributed to their lower crystallinity and disordered surface spins. The presence of some amount of oleic acid from the synthesis adsorbed on the surfaces can also account for this.

**Figure 12:**
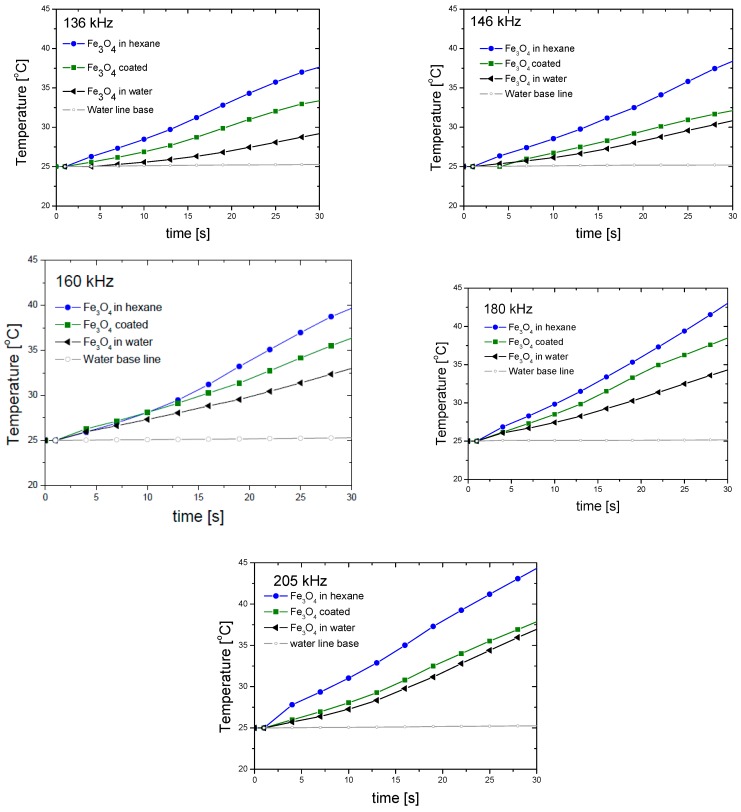
Heating curves for Fe_3_O_4_ nanoparticles in water and hexane before and after coating with PDMAEMA, measured at different frequencies.

Hyperthermia heating curves (measured at 200 Oe) were obtained at different frequencies for all the samples, showing a rapid increase of the temperature from 25 to 45 °C in less than one minute (Figure 12). Pure water baseline was added as reference, showing the negligible effect of Joule heating in the data. For all the frequencies tested, we can observe a noticeable decrease in the heating rate of bare Fe_3_O_4_ nanoparticles in water as compared to hexane, due to the lower heat capacity of this solvent. In addition, we have discussed above that the transfer to water brings about an increase in size, and it can be estimated [25,30,39,40] that for the smallest particles (bare magnetite in hexane) both the Brownian (the thermal energy is delivered through the relative movement between the nanoparticle and the surrounding fluid) and Néel (the magnetic moment rotates in the particle, while the particle itself remains fixed) relaxations contribute to the heating, whereas only the former is expected for larger magnetite particles. However, after PDMAEMA coating, the Fe_3_O_4_ nanoparticles become stable in aqueous medium and the hyperthermia response is clearly increased (Figure 12) in all cases.

**Figure 13:**
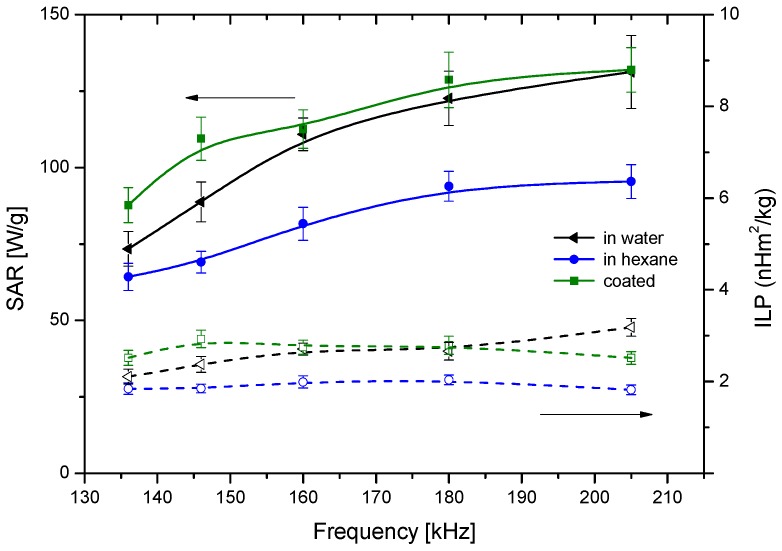
Specific absorption rate (SAR) (solid lines) and intrinsic loss power (ILP) (dotted line) values of the Fe_3_O_4_ nanoparticles in water, in hexane and coated with PDMAEMA in water.

A more quantitative understanding of the relative efficiencies of the different particles can be obtained by evaluating both SAR and ILP (Figure 13). In general, both quantities are almost frequency independent for the explored range, and the fact that the coated particles behave almost ideally is confirmed by the proximity of their corresponding SAR values to those of bare magnetite in water [41]. Interestingly, the best particles regarding hyperthermia are precisely the polymer-coated ones: it is likely that the relaxation of the individual particles inside the polymer envelope will add to the effect of coated nanostructures relaxation. In addition, the thermosensitive response of PDMAEMA seems to help in this task: the shrinking of the polymer may make the matrix more rigid, increasing the friction of the MNPs inside it, and producing further heating. The SAR values found in this work for the thermosensitive polymer-coated magnetite are similar to those reported by Purushotham et al. [42]. Regarding ILP, its values are also similar to those reported in the literature [41], although Lahiri et al. [43] have found exceedingly high (above 100 nH·m^2^·kg^−1^) data with suspensions of phosphate-coated magnetite.

## 5. Conclusions

Homogeneous, spherical polymer-coated magnetite nanoparticles have been successfully prepared, showing a superparamagnetic response of the MNPs synthesized. Particle size dependence with temperature was studied for bare and coated MNPs. Polymer coating has been demonstrated to provide extra stability of the MNPs in aqueous media, maintaining the particle size close to 100 nm in a pH range of 4–12. The organic/magnetic composition of the functionalized nanoparticles was calculated, obtaining a 74 wt % magnetic component, which was enough to supply an effective heating capability for magnetic hyperthermia response. A large SAR value was obtained for all the samples, especially the PDMAEMA-coated MNPs revealing an enhanced heating capacity as compared to bare MNPs. This thermo-sensitive response is essential to realize the therapeutic potential of hyperthermia applications, where the applied field amplitude and frequency are limited by practical and clinical considerations.
